# Microbial habitat connectivity across spatial scales and hydrothermal temperature gradients at Guaymas Basin

**DOI:** 10.3389/fmicb.2013.00207

**Published:** 2013-07-25

**Authors:** Stefanie Meyer, Gunter Wegener, Karen G. Lloyd, Andreas Teske, Antje Boetius, Alban Ramette

**Affiliations:** ^1^HGF-MPG Joint Research Group on Deep Sea Ecology and Technology, Alfred Wegener Institute for Polar and Marine ResearchBremerhaven, Germany; ^2^HGF-MPG Joint Research Group on Deep Sea Ecology and Technology, Max Planck Institute for Marine MicrobiologyBremen, Germany; ^3^MARUM Center for Marine Environmental Sciences, University of BremenBremen, Germany; ^4^Department of Microbiology, University of TennesseeKnoxville, TN, USA; ^5^Department of Marine Sciences, University of North Carolina - Chapel HillChapel Hill, NC, USA

**Keywords:** microbial habitat connectivity, bacterial diversity, Guaymas Basin, ARISA

## Abstract

The Guaymas Basin (Gulf of California) hydrothermal vent area is known as a dynamic and hydrothermally vented sedimentary system, where the advection and production of a variety of different metabolic substrates support a high microbial diversity and activity in the seafloor. The main objective of our study was to explore the role of temperature and other environmental factors on community diversity, such as the presence of microbial mats and seafloor bathymetry within one hydrothermally vented field of 200 × 250 m dimension. In this field, temperature increased strongly with sediment depth reaching the known limit of life within a few decimeters. Potential sulfate reduction rate as a key community activity parameter was strongly affected by *in situ* temperature and sediment depth, declining from high rates of 1–5 μmol ml^−1^ d^−1^ at the surface to the detection limit below 5 cm sediment depth, despite the presence of sulfate and hydrocarbons. Automated Ribosomal Intergenic Spacer Analysis yielded a high-resolution fingerprint of the dominant members of the bacterial community. Our analyses showed strong temperature and sediment depth effects on bacterial cell abundance and Operational Taxonomic Units (OTUs) number, both declining by more than one order of magnitude below the top 5 cm of the sediment surface. Another fraction of the variation in diversity and community structure was explained by differences in the local bathymetry and spatial position within the vent field. Nevertheless, more than 80% of all detected OTUs were shared among the different temperature realms and sediment depths, after being classified as cold (*T* < 10°C), medium (10°C ≤ *T* < 40°C) or hot (*T* ≥ 40°C) temperature conditions, with significant OTU overlap with the richer surface communities. Overall, this indicates a high connectivity of benthic bacterial habitats in this dynamic and heterogeneous marine ecosystem influenced by strong hydrothermalism.

## Introduction

The Guaymas Basin is located in the central Gulf of California and represents a unique hydrothermal sedimentary basin. High-temperature fluids, which pass through an on average 100-m-thick sedimentary cover, facilitate the pyrolysis of buried organic matter (Simoneit and Lonsdale, 1982) and enrich hydrothermal sediments in petroleum-like compounds, light hydrocarbons (methane, organic acids) and ammonia (Von Damm et al., 1985; Bazylinski et al., 1988; Martens, 1990). The hydrothermal sediments are characterized by a wide range in temperature regimes, generally reaching the known limits of life (>120°C) at 15–40 cm below the sediment surface (McKay et al., 2012). The fluids discharged at Guaymas Basin contain hydrogen, carbon dioxide and hydrogen sulfide (Welhan and Lupton, 1987; Elsgaard et al., 1994; Paull et al., 2007). Supported by this large variety of potential microbial energy sources, Guaymas Basin sediments host diverse anaerobic and aerobic microbial communities (Teske et al., 2002). Among these are the giant sulfide oxidizers *Beggiatoa* that can form thick white and orange bacterial mats on the seafloor, in habitats characterized by high sulfide fluxes (Jannasch et al., 1989; Nelson et al., 1989; McKay et al., 2012). In benthic ecosystems, these mats are hence often used as visual indicators of biogeochemical hotspots of high hydrocarbon flux and chemosynthetic production (Van Gaever et al., 2006; Lichtschlag et al., 2010; Lloyd et al., 2010). The key microbial processes such as nitrification (Mével et al., 1996; Baker et al., 2012), nitrate reduction (Bowles et al., 2012), sulfate reduction (Elsgaard et al., 1994; Weber and Jørgensen, 2002; Dhillon et al., 2003) and methanogenesis (Dhillon et al., 2005; Teske, 2010), were found to occur across a wide range of temperatures, suggesting a high diversity of the main functional groups of bacteria and archaea. Archaeal-bacterial consortia that mediate the anaerobic oxidation of methane (AOM) with sulfate are an important microbial community component in the Guaymas hydrothermal sediments (Teske et al., 2002, 2003), but *ex-situ* measurements of AOM have shown that this process is limited to temperatures <80°C (Kallmeyer and Boetius, 2004). Previous studies indicated that different types of methanotrophs are favored by different temperature ranges (Holler et al., 2011; Biddle et al., 2012).

Few studies have investigated in detail spatial distribution patterns of microbial communities at Guaymas Basin (e.g., Guezennec et al., 1996; Edgcomb et al., 2002; Kysela et al., 2005), to understand the main drivers of microbial diversity. Microbes are known to display biogeographic patterns, ranging from cosmopolitanism to provincialism, but the underlying mechanisms that generate and maintain those patterns at a wide range of spatial scales remain largely underexplored (Hughes Martiny et al., 2006; Ramette and Tiedje, 2007a; Zinger et al., 2011; Hanson et al., 2012). Within the conceptual framework of metacommunity dynamics (Leibold et al., 2004), and by statistically disentangling the effects of space and environment on community composition, insights into community assembly mechanisms such as patch dynamics, species sorting, mass effects or neutral processes, or combination thereof, may be obtained and quantified (Cottenie, 2005).

In this study, a high-resolution sampling effort of hydrothermal sediments was conducted, investigating the patchiness of bacterial communities at spatial scales ranging from decimeters to hundreds of meters, and across temperature ranges typical for psychrophilic (*T* < 10°C), mesophilic (10°C ≤ *T* < 40°C) and thermophilic (*T* ≥ 40°C) communities. Community fingerprinting data were obtained by Automated Ribosomal Intergenic Spacer Analysis (ARISA), which is useful to describe variations in bacterial community structure at a higher genetic resolution than what is provided by 16S rRNA gene sequencing (e.g., Brown et al., 2005; Nocker et al., 2007), because it is based on the amplification of the more variable ITS (Internal Transcribed Spacer) region. It may thus help reveal core communities and community shifts of the dominant bacterial types (Nocker et al., 2007; Fuhrman et al., 2008), but see Ramette (2009) for the possible detection of minor populations. To test for a potential effect of niche differentiation by temperature regimes on Guaymas Basin benthic bacterial communities, the following variables were chosen: *in situ* temperature was assessed during sampling, and vertical profiles of microbial assemblages inhabiting different temperature ranges were compared. Spatial variation was additionally assessed by latitude, longitude and water depth, relating to seafloor landscape features such as hydrothermal mounds. Further environmental parameters included bacterial mat presence/color as potential indicators for habitat heterogeneity, e.g., with regard to sulfide fluxes (Grünke et al., 2012; McKay et al., 2012). To infer potential changes in microbial abundance and function, microbial cell numbers, and potential sulfate reduction rates were included as additional parameters.

## Materials and methods

### Sample collection

Sediment samples were obtained from a hydrothermally active field of 0.05 km^2^ in the Southern Guaymas trench (Gulf of California, ~2000 m water depth, 27°00.37′N to 27°00.49′N and 111°24.58′W to 111°24.44′W), by push coring into enclosed plastic tubes with the *Alvin* submersible (operated by Woods Hole Oceanographic Institution, Woods Hole, MA) during R/V *Atlantis* expedition AT15-40 in December 2008. *In situ* subsurface temperatures were measured before coring (<50 cm away from the sampled areas) by using either the external “High Temperature Probe” or the external “Heatflow Probe” on the *Alvin* submersible (operated by WHOI; for probe details see McKay et al., 2012). The temperature values used in this study are reported in Table S1. Replicate sediment cores were collected separately for bacterial community analyses, for sulfate reduction rate measurements and for pore water geochemistry. During ascent of the submersible, all cores were tightly closed and stored upright in a fixed position. Cores with intact layering of mats and sediments were used for further analyses. Subsampling of the cores occurred within 4–12 h after sampling and storage at 4°C. The upper 10–30 cm of sediment cores were sectioned into 1-cm and 2-cm horizons and were preserved for subsequent analyses accordingly (see below). Samples for molecular work were immediately frozen at −20°C.

### Determination of sulfate concentrations and sulfate reduction rates

For a limited number of cores, pore water was extracted by centrifugation of sediment from the respective horizons, and sulfate pore water concentrations were measured as described previously (Biddle et al., 2012; McKay et al., 2012). Additional cores were used to measure sulfate turnover constants *ex situ* using the whole-core injection method (Jørgensen, 1978) with 5–10 μl carrier-free ^35^SO^2−^_4_ (dissolved in water, 50 kBq) in the dark, at a fixed temperature of 20°C for all cores. After 8–24 h incubation time, samples were preserved in 20 ml of 20% (w/v) ZnAc solution. Sulfate turnover constants (Table S2) and average sulfate concentrations (Table S3) determined in adjacent cores of the same dive were used to calculate potential sulfate reduction rates (Kallmeyer et al., 2004; Felden et al., 2010). Because of the substantial heterogeneity of *in situ* temperature and pore water composition between cores a few centimeters or decimeters apart (McKay et al., 2012), the resulting potential sulfate reduction rates (Table S2) should be regarded as an approximation. To show that sulfate concentrations are variable even over small spatial scales, the measured sulfate turnover constants are plotted alongside sulfate concentrations for nearby cores in Figure S1.

### Acridine orange direct cell counting (AODC)

Sediment sections (2 ml) for microbial cell counts were fixed onboard in 4% formaldehyde/seawater (9 ml) and stored at 4°C. In the home laboratories, samples were stained with acridine orange according to a modified protocol (Boetius and Lochte, 1996) of Meyer-Reil (1983). For each sample, single cells were counted on at least 2 replicate filters and for a minimum of 30 random grids per filter (dilution factors 2000–4000).

### Bacterial community fingerprinting

Sediment sections for DNA analyses were directly transferred to sterile plastic tubes and were kept frozen at −20°C until further use in the home laboratories. Total community DNA was extracted from 1 g of sediment by using the Ultra Clean Soil DNA Isolation Kit (MoBio, Carlsbad, CA) and by following the manufacturer's specifications for maximum yield. DNA was eluted in 100 μl 1 × TE buffer (Promega Corporation, Madison, WI) and stored at −20°C until further use. DNA concentration per volume buffer was measured by using a NanoQuant infinite M200 (Tecan, Crailsheim, Germany). Bacterial community analyses are based on profiles generated by Automated Ribosomal Intergenic Spacer Analysis (ARISA). The procedure has been previously published in detail (Ramette, 2009). In this study, a slightly modified version of the protocol was used: Within a 50 μl-reaction, final concentrations of PCR ingredients were 0.4 μM of each primer (Biomers, Ulm, Germany), 250 μM of each dNTP (peqGOLD Kit; Peqlab, Erlangen, Germany), 0.1 mg ml^−1^ BSA (Sigma-Aldrich Biochemie GmbH, Hamburg, Germany), 1 × Buffer S with 1.5 mM MgCl_2_ (Peqlab), 1.0 mM extra MgCl_2_ (Peqlab) and 2.5 U peqGOLD *Taq*-DNA-Polymerase (Peqlab). Per reaction, 20–25 ng of extracted DNA were used as template. Primers were ITSF (FAM-5′-GTC GTA ACA AGG TAG CCG TA-3′ Cardinale et al., 2004) and ITSReub (5′-GCC AAG GCA TCC ACC-3′ Cardinale et al., 2004).

### Statistical analyses

Quality assessment of ARISA profiles and binning were done as described in Ramette (2009). For merging PCR replicate profiles into master profiles, Operational Taxonomic Units (OTUs) consisted of peaks that occurred at least once among the respective PCR triplicates. All numerical analyses were conducted in R (v.2.13.2; The R Foundation for Statistical Computing; www.R-project.org) using the *vegan* library as well as standard packages and custom scripts (available at www.ecology-research.com).

Contextual parameters are summarized in Tables S1 and S4. DNA concentrations and total cell numbers were log-transformed to ensure normal distribution of the data. Presence and absence of mat as well as mat color (i.e., white, orange and yellow vs. no mat) were recoded as dummy variables (Ramette, 2007). Numbers of shared OTUs between different categories of contextual parameters were calculated by first merging all respective profiles of a given category into a single community profile where all peaks present were counted. Non-metric Multidimensional Scaling (NMDS) was used to represent dissimilarity matrices based on Bray-Curtis or Jaccard coefficients into a reduced space (Legendre and Legendre, 1998). Redundancy Analysis (RDA) models were combined with stepwise selection procedure, so as to retain the minimum number of significant variables as indicated by minimal Akaike Information Criterion (AIC) model value. Different sets of explanatory variables were then tested for significance in a variation partitioning framework (Borcard et al., 1992; Ramette, 2007). When negative *R*^2^ values were obtained, as it is the case sometimes for partial regression models (Legendre and Legendre, 1998), they were set to 0, and the total explained variation was recalculated, by adding up only the positive fractions. The heterogeneity of bacterial community structures among and between samples associated to certain environmental categories was quantified by their average variation in bacterial beta-diversity to each category's centroid (e.g., Zinger et al., 2011) and tested for significance by ANOVA followed by Tukey's Honestly Significant Difference tests, as implemented in the R package *vegan*.

## Results

### Site description

**Figure 1:**
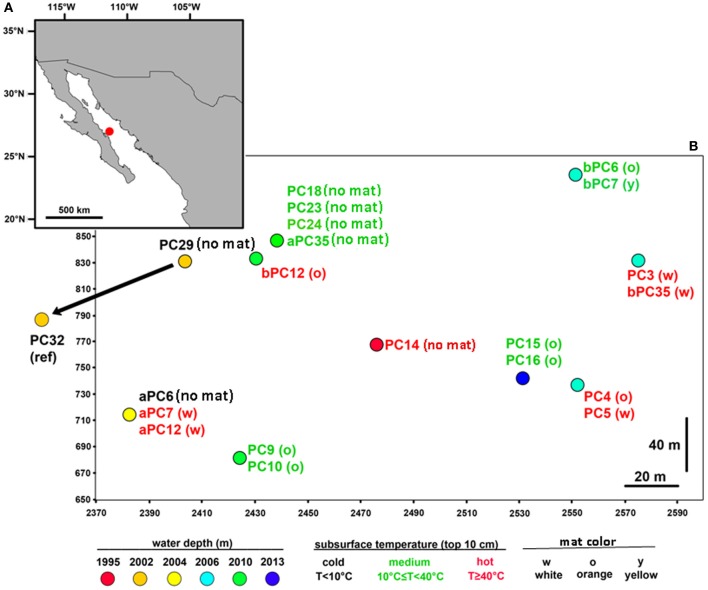
**Map of the sampled hydrothermal vent field. (A)** Location of the vent field in the Guaymas Basin (Gulf of California, ~2000 m water depth); **(B)** In total, 21 sediment cores were retrieved from an area of 200 × 250 m, including mat-covered and mat-free sediments. One non-hydrothermal sediment core (PC32) was sampled outside of the depicted area in the direction of the arrow. The map was generated in ArcMap (ArcGIS Desktop 9.3) with country boundaries obtained from www.geocommons.com. Positional coordinates (on a meter grid scale) are represented by ship- and submersible-fix values (see Table S2).

**Figure 2:**
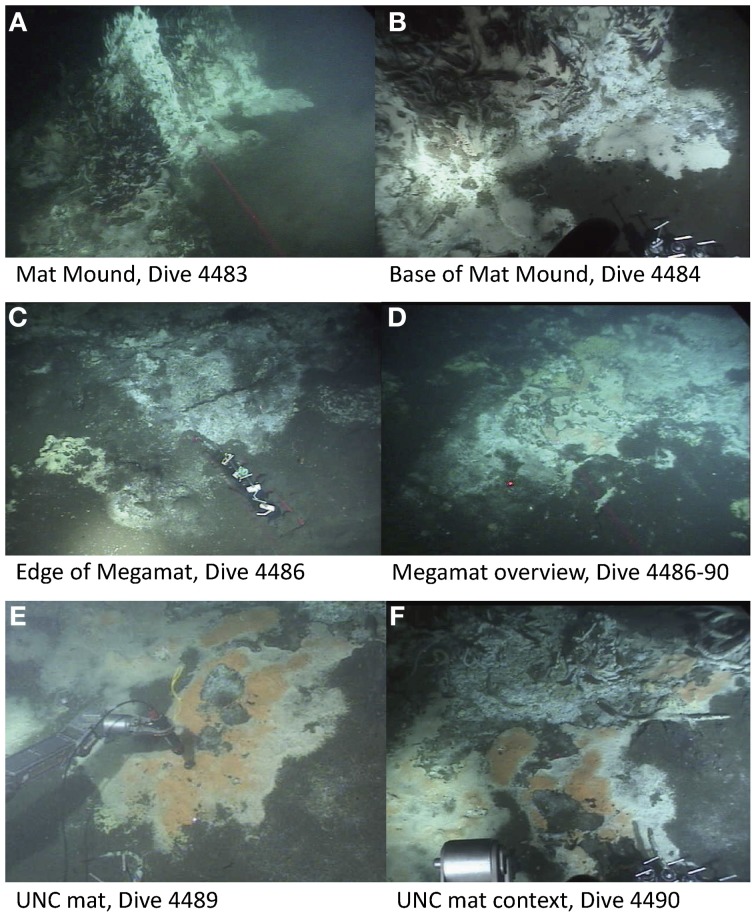
**Selected sampling sites of the Southern Guaymas trench**. This area is characterized by the occurrence of *Riftia* around hydrothermal mounds such as Mat Mound **(A,B)** and dense bacterial mats of white, orange or yellow color that are mainly formed by the giant sulfide oxidizer *Beggiatoa* on the sediment surface, for example at Megamat **(C,D)** and the UNC mat **(E,F)**. Image source: Woods Hole Oceanographic Institution, Woods Hole, MA.

To gain insight into the processes generating and maintaining bacterial community structure at Guaymas Basin, 21 sediment cores were obtained across an area of 200 × 250 m, including more or less hydrothermally vented sediments, and one background core was retrieved from outside the vent field (Figure 1; Table 1). The sampling area was located within a hilly area of the Southern Guaymas trench (Gulf of California) covering water depths of 1995–2013 m, and harboring hydrothermal mounds densely populated by *Riftia* tubeworms, sulfide spires and flanges as well as numerous white, orange and yellow *Beggiatoa* mats on the seafloor, some of them growing on mounds. One of these mounds, termed “Mat Mound” (N27°00.388; W111°24.560) was surrounded by an apron of hydrothermally active sediments overgrown with microbial mats, and was repeatedly visited for sampling (Figures 2A,B). Sediment pushcores aPC7, aPC12 and aPC6 (Figure 1) and several sulfate reduction cores (Figure S1) were sampled at this location. Bacterial mats growing on sediment surfaces typically had diameters of 1–2 m and could be several cm thick. One large mat-covered hydrothermal sediment area termed “Megamat” of ca. 10–15 m diameter (N27°00.464; W111°24.512) was visited and sampled repeatedly (Figures 2C,D); the subsurface temperature field underlying a portion of this extensive hydrothermal area was mapped and reconstructed in 3D (McKay et al., 2012). Pushcores PC18, PC23, PC24, and aPC35 (Figure 1) and several sulfate reduction cores (Figure S1) were sampled on the edge of Megamat. A smaller, orange-colored microbial mat termed UNC Mat (N27°00.445, W111°24.530) ca. 20 m southwest of Megamat was sampled (Figures 2E,F). This location showed strong *in situ* evidence for high-temperature sulfate-dependent methane oxidation (Biddle et al., 2012). Here sediment core bPC12 (Figure 1) and several sulfate reduction cores (Figure S1) were sampled. Additional sampling sites were visited, several of them on extensive survey dives (Alvin dives 4492 and 4493) to maximize the geographic range of sediment cores (Figure 1, Table 1).

**Table 1:** **Cores analyzed in this study by ARISA**.

**Label**	**Date**	**Dive**	**Water depth (m)**	**Location**	**Core description**
aPC12	06.12.08	4483	2004	Mat mound	Gray sediment with dark green and brown spots in between; white mat
aPC7	07.12.08	4484	2004	Mat mound	Gray sediment; white mat
aPC6	09.12.08	4485	2004	Outside mat mound	Green-gray sediment, worms; no mat
PC23	10.12.08	4486	2010	Outside Megamat	Olive-green sediment, gas holes; no mat
PC18	10.12.08	4486	2010	Outside Megamat	Top 5 cm fluffy brown, then olive-green sediment, gas holes; no mat
PC24	10.12.08	4486	2010	Outside Megamat	Top dark olive-green, rest olive-green, worm carcasses; no mat
bPC12	14.12.08	4489	2010	UNC mat, near Megamat	Top 5 cm fluffy, then olive sediment, hole at bottom, sulfidic; orange mat with few white filaments
aPC35	15.12.08	4490	2010	Megamat	Top 9–10 cm blackish, then olive-gray, gas holes, oily spots; no mat
PC32	16.12.08	4491	2002	100 m from Megamat	Top 1–3 cm fluffy blackish sediment, then mixed dark-gray with green-brown sediment; no detectable hydrothermal temperature gradient; no mat
PC29	16.12.08	4491	2002	50 m from Megamat	Top 1–3 cm fluffy brown sediment, then olive-gray sediment; no detectable hydrothermal temperature gradient; no mat
PC3	17.12.08	4492	2006	Survey site 1	Bubbling, top 2–4 cm brownish fluffy, then oily layer with oil/gas pockets, then grayish sediment, worm; white mat
PC4	17.12.08	4492	2006	Survey site 2	Bubbling, 9 cm organic layer, oily, with gas/oil pockets, then grayish sediment; orange mat
bPC6	17.12.08	4492	2006	Survey site 3	Top 2 cm fluffy, then 5 cm blackish, then olive sediment; orange mat with yellow parts
bPC35	17.12.08	4492	2006	Survey site 1	Fluffy brownish layer (partly pushed to bottom of the core), then grayish sediment with darker spots in-between, core liner melted at the bottom; white mat
PC5	17.12.08	4492	2006	Survey site 2	Bubbling, 1 cm gray sediment on top, then 4 cm grayish-brownish fluff, then 4 cm olive and oily layer with gas/oil pockets, then grayish sediment (more compact); white mat
bPC7	17.12.08	4492	2006	Survey site 3	Top 4 cm fluffy, then 4.5 cm darker and then brown-olive sediment; yellow mat
PC9	18.12.08	4493	2010	Survey site 4	8 cm grayish sediment, then mixed layer with red oily droplets, white inclusions towards the bottom; orange mat
PC10	18.12.08	4493	2010	Survey site 4	4 cm fluffy, then mixed sediment, some white inclusions, holes, red oily droplets; orange mat
PC14	18.12.08	4493	1995	Survey site 5	Top 6 cm darker, then 12 cm greenish, then 8 cm grayish sediment; no mat
PC15	18.12.08	4493	2013	Survey site 6	Olive sediment with blackish spots at top; orange mat
PC16	18.12.08	4493	2013	Survey site 6	3 cm fluffy, then olive-grayish sediment, white deposits, holes; orange mat

The distribution of mats across the overall investigated sampling area was patchy, and there was no obvious spatial gradient in the temperature field or in the distribution of bacterial mats recorded. As *Beggiatoa* mats are known to indicate geochemical and biodiversity hotspots (Lloyd et al., 2010; Grünke et al., 2012; McKay et al., 2012), they were repeatedly sampled within this study, resulting in the recovery of 13 mat-covered cores and 8 mat-free cores for comparison. Upon recovery, most cores were found to be rich in methane gas (Biddle et al., 2012; McKay et al., 2012). *In situ* subsurface temperatures varied between 3 and 96°C in the upper 10 cm of sediment (Table S1), and cores were classified into different temperature ranges according to these measurements.

**Figure 3:**
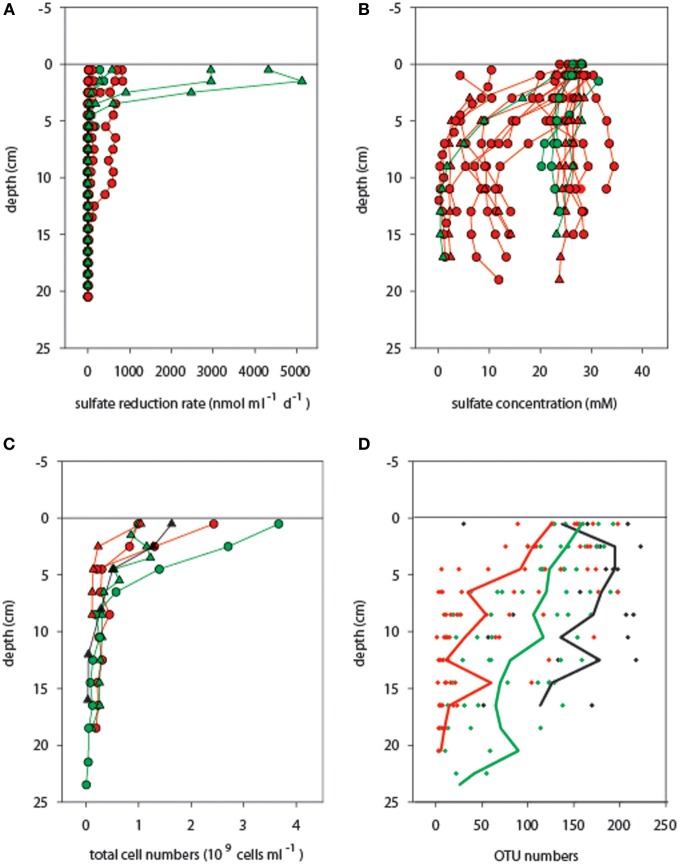
**Activity and biomass of microbial communities associated with different temperature ranges. (A)** Potential sulfate reduction rates and **(B)** sulfate concentrations were determined in adjacent cores (not available for cold cores). Total single cells **(C)** and OTU numbers **(D)** generally decreased with increasing sediment depth [the lines in **(D)** indicate average OTU numbers across cores]. Temperature categories indicated by color codes are cold (*T* < 10°C, black symbols), medium (10°C ≤ *T* < 40°C, green symbols), and hot (*T* ≥ 40°C, red symbols), as measured in the upper 10 cm of sediment before coring. Circles and triangles correspond to mat-covered and mat-free sediments, respectively.

Potential microbial sulfate reduction rates assessed at 20°C reached values as high as 5500 nmol ml^−1^ d^−1^ in the surface layers originating from an *in situ* temperature range of 3–40°C (Table S2), but then decreased abruptly beneath 5 cm sediment depth (*in situ* T approx. 10–40°C), and were almost absent below 10 cm (*in situ T* range 20–96°C) (Figure 3A, Table S1). In most cores sulfate was not depleted within the top 10 cm, indicating that availability of electron donors or sulfate did not limit sulfate reduction (Figure 3B; Table S3). This observed predominance of mesophilic sulfate reduction in surficial sediment layers may partially be explained by the fact that all potential sulfate reduction rate measurements were conducted at 20°C; thus, the contribution of high-temperature sulfate reduction rates to overall sedimentary sulfate-reducing activity was not assessed in this study. However, cell numbers also decreased rapidly with increasing sediment depth (Figure 3C; Table S4), varying between 1.0 and 3.7 × 10^9^ cells ml^−1^ in mat-covered and mat-free surface sediments, and declining to < 0.6 × 10^9^ cells ml^−1^ in sediment layers below 5 cm.

### Evaluation of environmental factors affecting community structure and function

Indicator factors for habitat variation tested within this study included *in situ* seafloor temperature (T), mat color/presence (MC) and sediment depth (SD), as well as bathymetry (measured as water depth, WD), and coordinates in the local sampling grid in meters (X,Y) as defined by seafloor radiobeacons (pingers) set out by RV *Atlantis* before the start of the sampling campaign (Table S4). Linear (Pearson) and rank-based (Spearman) correlations were used to determine the degree of correlation between all numerical parameters for the most complete subset of the data, which included *T* (real temperature values with number of samples *n* = 46; Table S1), SD, WD, X and Y. The analyses revealed significant positive correlations between SD and *T* (Pearson's *r* = 0.653, *P* < 0.001), with the deeper sediment layers representing hotter habitats (Table S1), as well as between X and Y sampling grid coordinates (Pearson's *r* = 0.420, *P* < 0.01), reflecting the fact that most of the sampling took place within a specific area, and not randomly dispersed around the zero origin of the coordinates. All other pairwise comparisons among parameters (i.e., T, SD, WD, X and Y) were not significant. Mat color variation was found to be significantly related to bathymetry (WD; F ratio = 11.003, *P* ≤ 0.001; explaining 20% of the variation in mat color) and spatial distance (X + Y; F ratio = 8.135, *P* ≤ 0.001; 25% of explained variation). There was no significant relationship between MC and T, when using the subset of data for which contextual *in situ* temperatures were obtained (*n* = 46). However, when assigning the full data set including all sediment depths (*n* = 188) into three temperature categories (cold *T* < 10°C, medium 10°C ≤ *T* < 40°C, hot *T* ≥ 40°C, as measured in the upper 10 cm of sediment), *T* was significantly (*P* ≤ 0.001) related to MC and explained up to 28% of the observed variation. Within individual mat locations, orange mats were found in the central area (Figure 1) characterized by the steepest geochemical gradients, and inferred hydrothermal fluxes (McKay et al., 2012), and were associated with temperatures ranging from 4 to 96°C (average 33 ± 26.5 [sd] °C; Table S4).

### Microbial cell numbers and variation in OTU number

**Figure 4:**
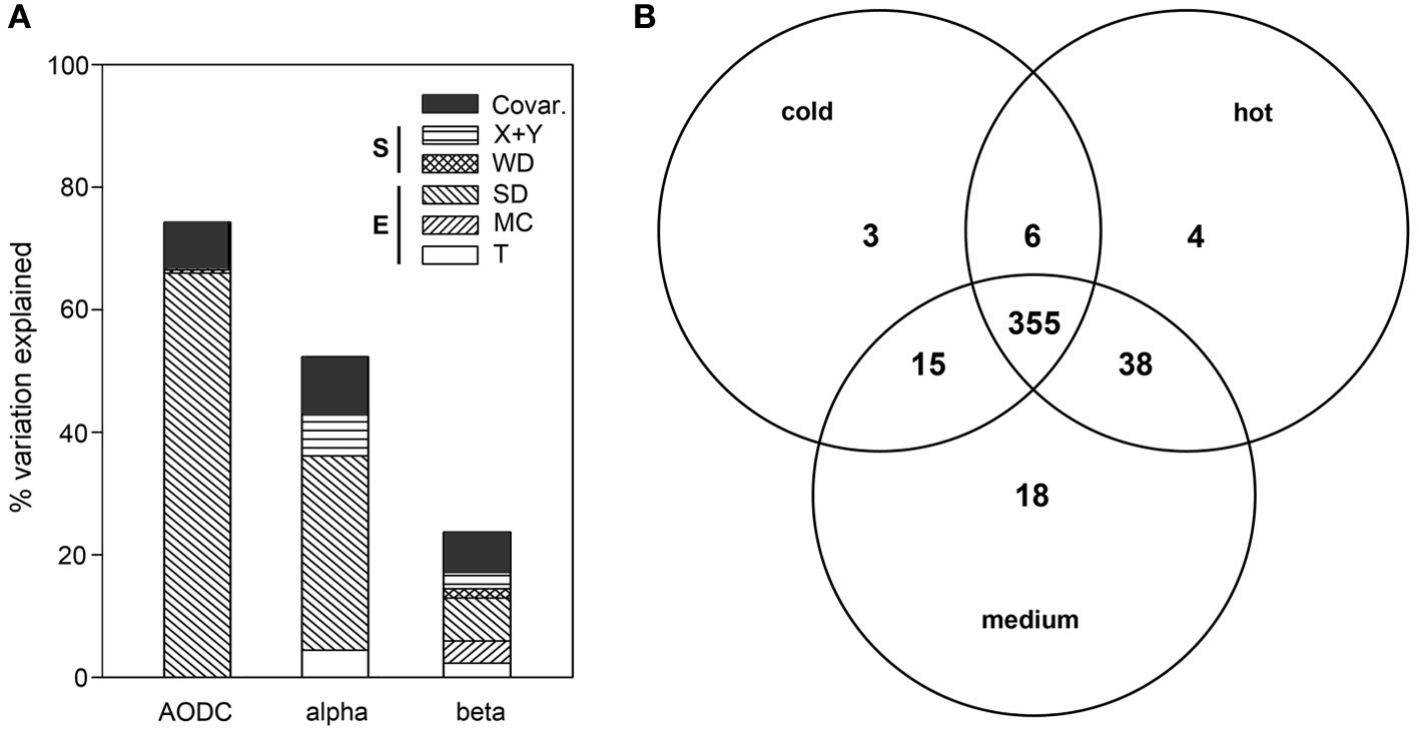
**(A)** Partitioning of the variation in total cell numbers (AODC), bacterial OTU numbers (alpha-diversity) and ARISA bacterial community structure (beta-diversity) as a function of environmental and spatial parameters. The plot depicts the percent explained variation by each significant contextual parameter. Environmental variables (E) included *T* (subsurface temperature), MC (mat presence/color) and SD (sediment depth). Spatial variables (S) included WD (water depth) as well as X + Y (spatial distance). **(B)** OTU partitioning according to the three temperature categories, namely cold (*T* < 10°C), medium (10°C ≤ *T* < 40°C) and hot (*T* ≥ 40°C), with *T* measured in the upper 10 cm of sediment.

Cell numbers declined significantly with sediment depth (partial coefficient = −0.161, *P* < 0.001). The overall variation in cell numbers (Figure 4A) was significantly explained by SD and WD (full model: 74%; *n* = 49), with SD alone explaining up to 66% of the observed variation (*P* < 0.001), while WD, respectively, explained 0.5% (*P* < 0.01; partial coefficient = 0.144, *P* < 0.001) of the variation in cell number (see Supplementary text and Figure S2, for analyses of the variation in extracted DNA concentrations).

Furthermore, also OTU number decreased with increasing temperature and sediment depth (Figure 3D). A total pool of 439 different OTUs was detected when considering all 188 samples, with OTU numbers per sample ranging from 1 to 223. OTU numbers were negatively related with SD (Figures S3, S4; Pearson's *r* = −0.567, *P* < 0.001; *n* = 188) and *T* (Pearson's *r* = −0.500, *P* < 0.001; *n* = 46, real temperature values), representing a substantial loss of bacterial richness with deeper, generally hotter sediments. Higher OTU numbers were obtained on average for the upper sediment layers (112 ± 65 OTUs; 0–10.5 cm) than for the deeper ones (54 ± 61 OTUs; 11–24 cm). However, considering sediment depth alone, maximum and minimum numbers of OTUs fell into a wide range, with 3–223 OTUs for the upper horizons and 1–218 OTUs for the deeper layers. The two depth categories had 90% OTUs in common.

Across all sediment depths, the range of OTU numbers determined for the different *T* categories (cold *T* < 10°C, medium 10°C ≤ *T* < 40°C, hot *T* ≥ 40°C) also varied widely, i.e., 30–223 OTUs (cold), 2–214 OTUs (medium) and 1–198 OTUs (hot), indicating a large spatial variability as well as potentially also a temporal variability of temperature (Figure 3D). The average OTU numbers decreased from cold to hot samples with 165 ± 60, 116 ± 59, and 90 ± 63 OTUs for cold, medium and hot samples, respectively. Cold and medium temperature samples shared 85%, cold and hot samples 86%, and medium and hot cores 90% of their OTUs (0–24 cm sediment depth). The reference core (PC32, cold), taken outside of the hydrothermal vent field, shared between 70 and 77% of its OTUs with any other cores taken within the surveyed area, while the percentages of shared OTUs was higher among samples within the vent field ranging from 83 and 90% (all *T* categories compared). Further OTU partitioning revealed that the number of unique OTUs in cold (3 OTUs) and hot habitats (4 OTUs) was lower than that of intermediate temperatures (18 OTUs) (Figure 4B).

Mat presence was generally associated with lower OTU numbers for the upper 10 cm of sediment, with average values of 93 ± 58 for mat-covered sediments vs. 149 ± 61 OTUs for mat-free sites (see also Figure S5), concurring with previous work suggesting that the sulfide- and methane-rich regime selects for a more specialized microbial community than in normal surface sediments (Lloyd et al., 2010). However, when taking all sediment depths into account, no clear difference in total OTU numbers between mat-covered (2–198 OTUs) and mat-free sites (1–223 OTUs) could be detected. The percentage of shared OTUs between mat-free and mat-covered sediments ranged from 63 to 88%. Multivariate analyses with the full data set (*n* = 188) indicated that variation in OTU number could be best explained by variation in SD (32%), space (X + Y; 7%) and *T* (4%), altogether explaining 52% of the total variation (Figure 4A).

### Changes in bacterial community structure

**Figure 5:**
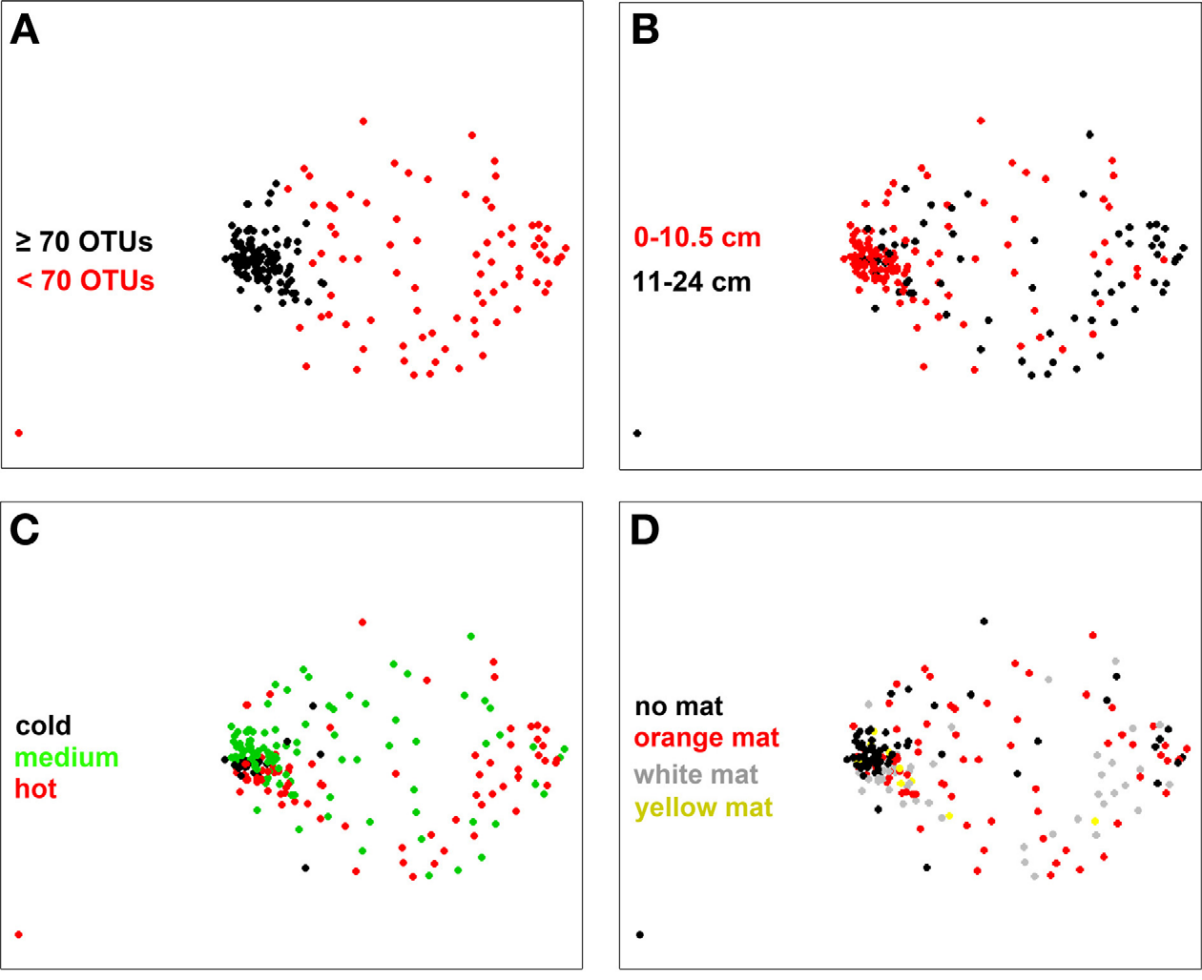
**NMDS plot of bacterial community structure based on a Bray-Curtis distance matrix (stress 0.16)**. Color coding was done according to **(A)** bacterial OTU number threshold (70 OTUs), **(B)** sediment depth, **(C)** subsurface temperature as categories cold (*T* < 10°C), medium (10°C ≤ *T* < 40°C) and hot (*T* ≥ 40°C, *T* measured in the upper 10 cm of sediment), and **(D)** mat color.

When considering all samples on the NMDS ordination plot (Figure 5) a clear separation appeared between samples that contained <70 OTUs and those with a higher richness (as determined by a frequency distribution analysis; Figure S6). Overall, samples with OTU numbers <70 OTUs were less similar to each other (i.e., communities were more heterogeneous) than the ones with OTU numbers higher than 70 OTUs (Figure 5A). They were mostly associated with sediment layers deeper than 10 cm and a temperature range of 20–96°C, depending on core and sampling location (Figure 5B; Table S1). Samples with OTU numbers ≥70 OTUs were mostly associated to cold (19 samples, i.e., 18% of all samples) or medium temperature (73 samples or 70%) conditions and generally originated from the top 10 cm surface layers (81 samples, i.e., 77% of all samples) (Figure 5C; Figure S7). No significant pattern was found to be associated with variation in mat color (Figure 5D), but within the group of samples with ≥70 OTUs, mat-free sediment samples were more similar to each other than mat-associated ones (Figure 5D).

When taking all contextual parameters analyzed here into account, 24% of the observed variation in bacterial community structure could be explained (Figure 4A; *n* = 188). Most of the variation was explained by SD (7%) and MC (4%), followed by spatial distance (X + Y, 3%), *T* (2%) and WD (2%), when taking the variations of each other parameter into account (partial regression analyses done via variation partitioning). All of these factors had highly significant (*P* ≤ 0.001) effects. Beta dispersion analyses revealed that medium temperature cores contained the highest community heterogeneity (average distance to centroid: 0.60), as compared to hot (average distance to centroid: 0.58), and cold cores (average distance to centroid: 0.46). Tukey's HSD tests indicated highly significant differences between cold cores and all other categories (*P* < 0.001), but no significant difference between hot and medium cores (*P* > 0.05).

## Discussion

### Impact of temperature on benthic bacterial communities at Guaymas basin

Guaymas Basin hydrothermal sediments are rich in hydrocarbons, methane, hydrogen sulfide, and contain a variety of other potential microbial energy sources, supporting complex anaerobic and aerobic microbial communities (Bazylinski et al., 1988; Martens, 1990; Dhillon et al., 2005; Biddle et al., 2012). Furthermore, the sediments at Guaymas Basin are characterized by a wide range of temperatures (3–200°C), thus crossing the realms of psychrophilic, mesophilic and thermophilic bacterial communities, as well as abiotic zones within small spatial scales (McKay et al., 2012). Temporal dynamics in fluid flow may also be high and can add substantial variation in fluxes of electron donors and acceptors as well as temperature (Gundersen et al., 1992). It is important to note that *in situ* temperatures obtained during sampling could rather be snapshots in time, and a site vented by very hot fluids a few days before may still show substantial disturbance effects after cooling, such as low cell counts and a low richness and sulfate reduction activity.

Guaymas Basin sediments are long known to be an interesting natural laboratory for the study of temperature-dependent oxidation of methane and sulfate reduction, and both processes have been found over a wide range of temperatures (Weber and Jørgensen, 2002; Kallmeyer and Boetius, 2004; Holler et al., 2011; Biddle et al., 2012). In general, at 20°C potential microbial sulfate reduction was strongly repressed below 4–5 cm (Figure 3A), despite the presence of sulfate and methane as well as other electron donors throughout the core, a phenomenon previously observed for Guaymas Basin sediments (Martens, 1990; Elsgaard et al., 1994; Weber and Jørgensen, 2002; Dhillon et al., 2005; Biddle et al., 2012; McKay et al., 2012).

Potentially, episodic heat pulses by upward advecting hot fluids may act as strong disturbances to the microbial assemblages selected by certain temperature ranges in space and time. Upward advection of very hot fluids >100°C could even lead to sterilization of the present assemblages, which then will be repopulated by the surface communities. This may explain the reduction or disappearance of biomass and diversity below 5–10 cm sediment depth detected in total cell numbers (Figure 3C), as well as bacterial OTU numbers (Figure S3). A substantial decline in cell numbers within a decimeter of the seafloor has also been observed in other highly fluid-flow advected marine sediments (Lösekann et al., 2007; Grünke et al., 2011), as well as in disturbed seep environments such as submarine mud volcanoes (Pop Ristova et al., 2012). If such geophysical disturbances can affect microbial abundances, they are also likely to affect community diversity and function. Even though archaeal diversity was not covered by our molecular approach, it is known for instance that archaea isolated from Guaymas Basin have demonstrated susceptibility to heavy metals (Edgcomb et al., 2004) or to the combination of low pH, high sulfide and low temperature typically present in vent fluids (Lloyd et al., 2005).

The rapid loss of OTU number and microbial activity with sediment depth and increasing temperature indicate that from a relatively diverse community, only few members can occupy the niches available in the deep hot sediments at Guaymas (Figures 3–5). Interestingly, most of these OTUs were not unique, but occurred throughout the surface sediments sampled from the vent field. Hence, it seems likely that the prevailing diverse communities of bacteria in the highly reduced surface sediments of Guaymas Basin are functioning as a seed bank to deeper depths, and that many community members are adapted to a relatively wide range of temperature conditions. This is in general accordance with findings on the relatively broad temperature ranges of anaerobic methane oxidizers and sulfate reducers at Guaymas (Elsgaard et al., 1994; Weber and Jørgensen, 2002; Kallmeyer and Boetius, 2004; Holler et al., 2011; Biddle et al., 2012). Furthermore, ANME1-Guaymas archaea were previously found at temperatures between 20 and >90°C, suggesting their eurythermal nature and adaptation to fluctuations in temperature and heat flow (Biddle et al., 2012). Most likely the small-scale variation in space and time of the upward advective transport of hot hydrothermal fluids, and downward mixing of seawater penetrating surficial sediments (Gundersen et al., 1992) is likely exerting a substantial challenge to the microbial community, leading to substantial losses in diversity with sediment depth, and blurring a typical temperature-induced zonation of microbial habitats in the sediments.

### Other niche effects on bacterial community composition

*Beggiatoa* mats are generally linked to steep gradients and high fluxes of sulfide, DIC and CH_4_; they can be flushed by oxygenated seawater (Gundersen et al., 1992) and indicate hotspots of CH_4_ and sulfur cycling (Lichtschlag et al., 2010; Lloyd et al., 2010; Grünke et al., 2012; McKay et al., 2012). Their distribution across the investigated area at Guaymas Basin was patchy and not directly related to bathymetry or temperature regimes. Thus, we investigated whether the sampled areas may represent hotspots of bacterial diversity, especially via the presence of *Beggiatoa* that have been proposed to be acting as ecosystem engineers at Guaymas Basin by “providing specialized habitats for unique assemblages of species, thereby creating seafloor biodiversity hotspots” (Levin and Dayton, 2009). OTU number was compared across different mat types and mat-free sediments. In the top 2.5 cm sediment layers, mat-covered sediments were associated on average with fewer OTU with 137 OTU (*n* = 30) as compared to 162 OTU (*n* = 14) in mat-free sediments (two-sample Wilcoxon test, *W* = 115, *P* = 0.0172). These numbers are comparable to those found in and around sulfide-oxidizer mats at cold seeps along the Norwegian continental margin (on average 121–166 OTUs in 0–2.5 cm sediment depth; Grünke et al., 2012). The total OTU number of 439 detected in Guaymas Basin sediments was similar to bacterial OTUs from a cold seep site at the West African margin (~3200 m water depth, 450 OTUs; Pop Ristova et al., 2012). The percentage of shared OTUs between mat-covered and mat-free sediments (63–88%, 0–24 cm) was slightly higher than what has previously been found in a study on bacterial sulfide oxidizer mats at the Norwegian margin (41–63%, 0–2.5 cm; Grünke et al., 2012), and for other chemosynthetic communities at the cold seep REGAB of the West African margin (average 74%, 0–10 cm; Pop Ristova et al., 2012). The effects on community diversity of mat presence and color (corresponding to different *Beggiatoa* spp.) (McKay et al., 2012), could not be further disentangled in this study, because of the highly variable temperature regimes within the different mats. Although mat-forming sulfide oxidizers of the family *Beggiatoaceae* have previously been shown to specifically associate with certain types of bacteria (Kojima et al., 2006; Prokopenko et al., 2006; Teske et al., 2009), our results overall did not support the idea that the presence *Beggiatoa* mats was associated with higher bacterial diversity or specificity as compared to neighboring mat-free sediment communities.

### Spatial effects on bacterial communities

The investigated hydrothermal field at Guaymas Basin was characterized by various structures typical for vent fields such as chimneys and mounds overgrown with *Beggiatoa* mats (Figure 2A), and dense *Beggiatoa* mats covering surface sediments (Figures 2B,C; Jannasch et al., 1989), vent chimneys and sulfide spires (Figures 2D–F). There was a slightly elevated area in the middle of the investigated area, where the water depth reached only 1995 m as compared to >2000 m in the other areas, however, this feature was not distinctly related to a temperature gradient. Nevertheless, we explored whether spatial distance and bathymetry (mounds vs. troughs on the landscape level) had an effect on community assemblage.

The complex interplay of environmental and spatial factors on microbial diversity has already been observed in patchy terrestrial environments (e.g., Ramette and Tiedje, 2007b). Our analyses of 0.05 km^2^ of the Guaymas hydrothermal field showed that most tested biological variables, i.e., total cell numbers, OTU numbers and beta-diversity, were significantly influenced by spatial factors (X, Y or WD) at the scale of meters to hundreds of meters (Figure 4A). Total cell numbers were positively correlated with bathymetry, so that elevated landscape features showed a lower microbial biomass (based on partial linear regression models), but they were not correlated with latitude and longitude. OTU numbers were correlated to the geographic positions of the samples (both increasing with Y and decreasing with X), but not to the bathymetric features. Changes in overall bacterial community structure could be related to changes in both bathymetry and geographic locations, yet we were unable to determine which environmental variables were explaining these spatial community patterns.

By comparing the effects of each investigated parameter, environmental factors (T, SD, MC) always explained more of biological variation than combined spatial variables (X, Y, WD), in total cell numbers, OTU number, and beta-diversity. This is consistent with a recently published review on the current knowledge about what processes influence the distribution of microbes, and the percentages of total explained variation in our study are comparable to the overall reported mean of 50% across studies (Hanson et al., 2012). It should be noted that, because not all selective environmental variables can be assessed in field studies, and especially their past temporal variation remains unknown, pure spatial effects on community structure may be potentially overestimated (Cottenie, 2005; Hanson et al., 2012). Interpreted within a metacommunity ecology framework (Leibold et al., 2004), our findings overall suggest that Guaymas bacterial communities are subjected to a combination of species sorting (i.e., to dispersal associated with niche differentiation) and of mass effect (i.e., dispersal effects through source-sink relationships).

## Conclusion

Guaymas Basin hydrothermal sediment bacterial communities displayed a high variation in community richness and activity on small spatial scales. Community activity, abundance and richness declined substantially with increasing temperature, indicating that only few microbial types of the core community of the investigated vent field were adapted to populate deep hot sediments at temperatures >10°C and higher (Figure S7). However, community similarity was high across the different temperature regimes and habitat types sampled, and across the entire range of temperature regimes—from normal deep-sea settings to the predicted limits of life >120°C—only few unique microbial types were detected. This is best explained by the scenario of connected bacterial habitats, where temporary disturbances by the upward expulsion of hot fluids can locally decrease cell activity, biomass and diversity, and where a repopulation occurs by a diverse, eurythermic and more stable surface community of the highly reduced Guaymas sediments. Besides temperature and sediment depth, the presence of microbial mats, local bathymetry and spatial orientation in the vent field also showed significant effects on community richness and composition, but due to the complexity of the vent field, no distinct spatial gradient was detected, indicative of active but chaotic upward venting in space and time. Bacterial habitats seemed highly interconnected across the investigated vent area, which may be a consequence of dynamically fluctuating temperatures and biogeochemical factors.

### Conflict of interest statement

The authors declare that the research was conducted in the absence of any commercial or financial relationships that could be construed as a potential conflict of interest.
