# Supplementary material for: Microbial habitat connectivity across spatial scales and hydrothermal temperature gradients at Guaymas Basin
Source: Front Microbiol. 2013 Jul 25;4:207. doi: 10.3389/fmicb.2013.00207 (PMC3723108; doi:10.3389/fmicb.2013.00207)
Supplement: Supplementary file 1 [file DataSheet1.PDF]

Supplementary Material to

Microbial habitat connectivity across spatial scales and hydrothermal temperature gradients at Guaymas Basin

Additional Results and Discussion

DNA concentrations

Even though ARISA was performed with standardized amounts of DNA, the DNA concentrations varied across samples and we investigated whether this variation was related to any environmental drivers. Extracted DNA concentrations per g of sediment varied between 0.2-77 ng μl^{-1} (SI-Table 4). Like other parameters of microbial abundance DNA decreased with sediment depth except in some hot cores, and Pearson and Spearman's correlations showed a negative correlation between SD and DNA concentration (Pearson's r = -0.366, $P<0.001$; n = 188). DNA concentration was significantly influenced by a combination of factors with could explain 51% of the observed variation (Fig. 4A), with MC (mats generally giving higher DNA yields) explaining 17% ($P\leq0.001$), SD explaining 16% ($P\leq0.001$) and space (X+Y) explaining 18% ($P\leq0.001$). However in some very hot cores high DNA concentrations were found although very low cell numbers were detected (Fig. SI-2). This indicates either the presence of large amounts of fossil DNA in these samples or the interference of DNA and other extracted compounds extracted from the hot sediments in the quantification via optical absorbance. Furthermore, OTU numbers were positively correlated with DNA concentrations (Fig. SI-4; Pearson's r = 0.413, $P<0.001$; n = 188) and with total cell numbers (Fig. SI-5; Pearson's r = 0.549, $P<0.001$; n = 49).

SI-Table 1 – Whole temperature profiles used in this study.

	Temperature profiles												
**Depth (cm)**	**T1**	**T2**	**T3**	**T4**	**T5**	**T6**	**T7**	**T8**	**T9**	**T10**	**T11**	**T12**	**T13**
-5	3		3										
0	16	10	3		3	58	3	6	7	4	4	10	10
2				23	14								
5			3										
8		28			16								
10	80	87	3			96	20	50	54	27	29	57	38
12		61		44	35								
15			3										
16		58											
18					45								
19.5		98											
20	81	103	3			104	45	76	127	54	59	96	85
22				65	63								
23		37											
25		105	3										
26					75								
27		39											
30	86	97	3			102	67	86	149	71	80	126	94
32				82	87								
33		36											
34.5		108											
35		29	3										
37		65			99								
40	94	141	3			104	84	90	164	78	90	131	83
42				101	109								
**Dive**	4483	4484	4485	4486	4486	4489	4490	4492	4492	4492	4493	4493	4493
**Core no.(s)**	12	7	6	23, 24	18	12	35	3, 35	4, 5	6, 7	9, 10	14	15, 16
**Instrument**	HF8	HF1-4, HT1-2	HF	HF10	HF5, HT1	HF9	HF8	HF2	HF3	HF4	HF3	HF5	HF6

Temperature data listed here were measured *in situ* during cruise AT15-40 (2008) and contain previously unpublished data as well as data published in McKay et al. (2012) and Biddle et al. (2012). Temperatures were measured using either the High Temperature Probe (HT) or the Heatflow Probe (HF) operated by the *Alvin* submersible (operated by WHOI; for probe details see McKay et al., 2012). If several measurements had been conducted at the same location, values determined with the probe(s) closest to the respective core are listed. If more than 1 instrument is listed, average values are presented. Positive depth values correspond to measurements within the sediment, while negative values refer to measurements above the sediment (in the water column). In statistical analyses only those temperature values were used for that all other data (contextual parameters, community profiles) were available (grey cells).

SI-Table 2 – Sulfate reduction rates measured in this study.

depth	4483 PC13	4483 PC9	4484 PC26A	4484 PC27A	4486 PC31A	4486 PC 32A	4486 PC 31B	4488 PC 28	4488 PC9	4489 PC27/3	4489 PC33/3	4489 PC13	4489 PC9	4490 PC 36/1	4490 PC36/2
(cm bsf)	Sulfate reduction rate (nmol ml^{-1} d^{-1})														
0.5	285	820	1262	190	42	18	32	37	69	664	95	7	17	4326	579
1.5	382	834	1726	343	43	5	27	9	95	602	142	1	3	5129	278
2.5	82	291	383	276	36	2	13	2	88	491	117	0	9	2474	81
3.5	40	52	36	219	11	0	3	0	6	611	71	0	4	601	32
4.5	30	49	15	97	3	0	1	0	4	573	65	0	0	70	13
5.5	173	125	33	49	1	0	0	0	1	411	28	15	0	33	0
6.5		6	88	20	0	0	0	0	0	148	21	0	3	14	0
7.5		0	22	16	0	0	0	0	0	102	19	0		1	0
8.5		0	5	8	0	0	0	0	0	22	19			0	0
9.5		0	2	2	0	0	0	0	0	7	10			0	0
10.5		0	0	2	0	0	0	0	0	4	5			0	0
11.5		0	0		0	0	0	0	0	0	6			0	0
12.5					0	0	0	0	0	0	8			0	0
13.5					0	0	0	0	0	0	5			0	0
14.5					0	0	0	0	0	0	3			0	0
15.5					0	0	0	0	0	0	2			0	0
16.5					0	0	0	0	0	0	0			0	0
17.5					0		0	0	0					0	0
18.5					0		0	0						0	0
19.5					0		0							0	0

SI-Table 3 – Sulfate concentrations determined in this study

depth	4483 PC17	4483 PC23	4483 PC24	4484 PC2	4484 PC3	4484 PC6	4484 PC10	4484 PC14	4486 PC13	4486 PC14	4488 PC5	4488 PC10	4489 PC10	4489 PC11	4490 PC12	4490 PC25	4490 PC26	4490 PC27
(cm bsf)	Sulfate concentration (mmol l^{-1})																	
0			26.6		26.9								25.4			26.3	23.8	28.1
0.5											10.4		25.8					
1	26.7	24.9	23.0	20.0	19.9	30.4	29.0		26.9			4.3		25.1	22.8	25.5	23.3	26.1
1.5								31.4					22.8					
2											9.7							
3	22.4	14.4	25.7	18.5	8.4	24.3	26.6		19.9	6.1		10.5	24.0	24.3	16.5	23.0	22.7	23.7
3.5											6.7							
4.5								25.7			4.3							
5	14.8	9.0	24.5	22.7	4.3	14.5	25.1		8.4	2.6		3.4	21.2	21.9	9.0	23.1		23.3
6											3.1							
7	13.6	8.5	26.4		5.4	10.3	17.6	20.8	4.3	2.1		1.2	28.4	22.8	5.0	22.6		23.3
8											0.8							
9		9.4	26.4		9.1	7.1	12.1	20.2	5.4	2.4		0.3	29.4		1.7	22.2		
10											1.1							
11		9.4	27.9		11.8	8.3	11.2		9.1	2.1		0.4	26.9	25.7	0.7	22.9		
12											0.1							
13		9.7			14.2	6.4	11.3		11.8	2.3		0.8	28.5	28.2	0.4	23.8		
14											1.6							
15		11.2				6.5	13.9		14.2	2.0		1.1	26.5	28.5	0.4			
17		13.3				7.5				2.4		1.3			1.0			
19						11.9												

SI-Table 4 – Spatial and environmental parameters used in this study (cruise AT15-40, 2008).

Dive	Core	Label	Water depth (m)	Sediment layers (cm)	Sediment depth (cm)	DNA concentration (ng µl^{-1})	Location	Coordinates original (x)	Coordinates original (y)	Coordinates corrected (X)	Coordinates corrected (Y)	Mat color	Temperature original (°C)	Temperature category	Single cells (10^9 cells ml^{-1})
4483	12	aPC12_1	2004	0-1	0.5	47.7	mat mound	2382	716	2382	716	w	16	3	0.9949
4483	12	aPC12_3	2004	2-3	2.5	14.1	mat mound	2382	716	2382	716	w		3	0.8303
4483	12	aPC12_5	2004	4-5	4.5	3.2	mat mound	2382	716	2382	716	w		3	0.3040
4483	12	aPC12_7	2004	6-7	6.5	2.7	mat mound	2382	716	2382	716	w		3	0.2768
4483	12	aPC12_9	2004	8-9	8.5	1.8	mat mound	2382	716	2382	716	w		3	0.4490
4483	12	aPC12_11	2004	10-11	10.5	3.9	mat mound	2382	716	2382	716	w	80	3	0.2495
4483	12	aPC12_13	2004	12-13	12.5	3.6	mat mound	2382	716	2382	716	w		3	0.3124
4483	12	aPC12_15	2004	14-15	14.5	6.6	mat mound	2382	716	2382	716	w		3	0.2196
4483	12	aPC12_17	2004	16-17	16.5	1.9	mat mound	2382	716	2382	716	w		3	0.2312
4483	12	aPC12_19	2004	18-19	18.5	1.2	mat mound	2382	716	2382	716	w		3	0.1950
4484	7	aPC7_1	2004	0-1	0.5	28.1	mat mound	2382	716	2382	717	w	10	3	
4484	7	aPC7_3	2004	2-3	2.5	40.3	mat mound	2382	716	2382	717	w		3	
4484	7	aPC7_5	2004	4-5	4.5	15.2	mat mound	2382	716	2382	717	w		3	
4484	7	aPC7_7	2004	6-7	6.5	77	mat mound	2382	716	2382	717	w		3	
4484	7	aPC7_9	2004	8-9	8.5	40.7	mat mound	2382	716	2382	717	w	28	3	
4484	7	aPC7_11	2004	10-11	10.5	42	mat mound	2382	716	2382	717	w	87	3	
4484	7	aPC7_13	2004	12-13	12.5	18.5	mat mound	2382	716	2382	717	w	61	3	
4484	7	aPC7_15	2004	14-15	14.5	4.9	mat mound	2382	716	2382	717	w		3	
4484	7	aPC7_17	2004	16-17	16.5	2.7	mat mound	2382	716	2382	717	w	58	3	
4484	7	aPC7_19	2004	18-19	18.5	27.2	mat mound	2382	716	2382	717	w		3	
4485	6	aPC6_1	2004	0-1	0.5	9	outside mat mound	2382	716	2382	719	no mat	3	1	
4485	6	aPC6_3	2004	2-3	2.5	6.4	outside mat mound	2382	716	2382	719	no mat		1	
4485	6	aPC6_5	2004	4-5	4.5	4.4	outside mat mound	2382	716	2382	719	no mat	(3)	1	
4485	6	aPC6_9	2004	7-9	8	3.3	outside mat mound	2382	716	2382	719	no mat		1	
4485	6	aPC6_13	2004	11-13	12	3.8	outside mat mound	2382	716	2382	719	no mat		1	
4485	6	aPC6_17	2004	15-17	16	1.9	outside mat mound	2382	716	2382	719	no mat	(3)	1	
4486	23	PC23_1	2010	0-1	0.5	2.3	outside Megamat	2438	847	2438	850	no mat		2	1.0028
4486	23	PC23_2	2010	1-2	1.5	1.1	outside Megamat	2438	847	2438	850	no mat		2	0.8554
4486	23	PC23_3	2010	2-3	2.5	1.4	outside Megamat	2438	847	2438	850	no mat	23	2	1.1548
4486	23	PC23_4	2010	3-4	3.5	2.9	outside Megamat	2438	847	2438	850	no mat		2	1.2268
4486	23	PC23_5	2010	4-5	4.5	7.9	outside Megamat	2438	847	2438	850	no mat		2	0.5287
4486	23	PC23_6	2010	5-6	5.5	7.6	outside Megamat	2438	847	2438	850	no mat		2	0.6361
4486	23	PC23_7	2010	6-7	6.5	10	outside Megamat	2438	847	2438	850	no mat		2	0.3429
4486	23	PC23_9	2010	8-9	8.5	4.9	outside Megamat	2438	847	2438	850	no mat		2	0.3054
4486	23	PC23_11	2010	10-11	10.5	6.5	outside Megamat	2438	847	2438	850	no mat		2	0.3041
4486	23	PC23_13	2010	12-13	12.5	15.6	outside Megamat	2438	847	2438	850	no mat	44	2	0.2776
4486	23	PC23_15	2010	14-15	14.5	6.8	outside Megamat	2438	847	2438	850	no mat		2	0.2551
4486	23	PC23_17	2010	16-17	16.5	3.1	outside Megamat	2438	847	2438	850	no mat		2	0.2623
4486	23	PC23_19	2010	18-19	18.5	3.2	outside Megamat	2438	847	2438	850	no mat		2	0.1184
4486	18	PC18_1	2010	0-1	0.5	16.6	outside Megamat	2438	847	2439	850	no mat	3	2	
4486	18	PC18_3	2010	2-3	2.5	27.3	outside Megamat	2438	847	2439	850	no mat	14	2	
4486	18	PC18_5	2010	4-5	4.5	2.7	outside Megamat	2438	847	2439	850	no mat		2	
4486	18	PC18_8	2010	6-8	7	1.9	outside Megamat	2438	847	2439	850	no mat	(16)	2	
4486	18	PC18_12	2010	10-12	11	4	outside Megamat	2438	847	2439	850	no mat	(35)	2	
4486	18	PC18_16	2010	14-16	15	6.1	outside Megamat	2438	847	2439	850	no mat		2	
4486	18	PC18_20	2010	18-20	19	4.1	outside Megamat	2438	847	2439	850	no mat	(45)	2	
4486	24	PC24_2	2010	0-2	1	7.2	outside Megamat	2438	847	2437	850	no mat	(23)	2	

4486	24	PC24_6	2010	4-6	5	1.6	outside Megamat	2438	847	2437	850	no mat		2	
4486	24	PC24_10	2010	8-10	9	1.2	outside Megamat	2438	847	2437	850	no mat		2	
4486	24	PC24_14	2010	12-14	13	0.4	outside Megamat	2438	847	2437	850	no mat	(44)	2	
4489	12	bPC12_1	2010	0-1	0.5	44.9	UNC mat near Megamat	2428	832	2428	832	o	58	3	
4489	12	bPC12_3	2010	2-3	2.5	9.1	UNC mat near Megamat	2428	832	2428	832	o		3	
4489	12	bPC12_5	2010	4-5	4.5	4.4	UNC mat near Megamat	2428	832	2428	832	o		3	
4489	12	bPC12_7	2010	6-7	6.5	47.5	UNC mat near Megamat	2428	832	2428	832	o		3	
4489	12	bPC12_9	2010	8-9	8.5	17.1	UNC mat near Megamat	2428	832	2428	832	o		3	
4489	12	bPC12_11	2010	10-11	10.5	55.8	UNC mat near Megamat	2428	832	2428	832	o	96	3	
4489	12	bPC12_13	2010	12-13	12.5	39.3	UNC mat near Megamat	2428	832	2428	832	o		3	
4489	12	bPC12_15	2010	14-15	14.5	13.4	UNC mat near Megamat	2428	832	2428	832	o		3	
4489	12	bPC12_17	2010	16-17	16.5	29.6	UNC mat near Megamat	2428	832	2428	832	o		3	
4489	12	bPC12_19	2010	18-19	18.5	6	UNC mat near Megamat	2428	832	2428	832	o		3	
4490	35	aPC35_1	2010	0-1	0.5	16.4	Megamat	2438	847	2438	847	no mat	3	2	
4490	35	aPC35_3	2010	2-3	2.5	5.2	Megamat	2438	847	2438	847	no mat		2	
4490	35	aPC35_5	2010	4-5	4.5	7.2	Megamat	2438	847	2438	847	no mat		2	
4490	35	aPC35_7	2010	6-7	6.5	1.9	Megamat	2438	847	2438	847	no mat		2	
4490	35	aPC35_9	2010	8-9	8.5	3.4	Megamat	2438	847	2438	847	no mat		2	
4490	35	aPC35_11	2010	10-11	10.5	3	Megamat	2438	847	2438	847	no mat	20	2	
4490	35	aPC35_13	2010	12-13	12.5	6.8	Megamat	2438	847	2438	847	no mat		2	
4490	35	aPC35_15	2010	14-15	14.5	3.8	Megamat	2438	847	2438	847	no mat		2	
4490	35	aPC35_17	2010	16-17	16.5	5.6	Megamat	2438	847	2438	847	no mat		2	
4490	35	aPC35_19	2010	18-19	18.5	0.8	Megamat	2438	847	2438	847	no mat		2	
4490	35	aPC35_21	2010	20-21	20.5	0.7	Megamat	2438	847	2438	847	no mat	45	2	
4491	32	PC32_1	2002	0-1	0.5	19.2	100 m from Megamat	2354	807	2354	807	ref		1	
4491	32	PC32_3	2002	2-3	2.5	13.6	100 m from Megamat	2354	807	2354	807	ref		1	
4491	32	PC32_5	2002	4-5	4.5	11.5	100 m from Megamat	2354	807	2354	807	ref		1	
4491	32	PC32_7	2002	6-7	6.5	13.8	100 m from Megamat	2354	807	2354	807	ref		1	
4491	32	PC32_9	2002	8-9	8.5	7.8	100 m from Megamat	2354	807	2354	807	ref		1	
4491	32	PC32_11	2002	10-11	10.5	16.8	100 m from Megamat	2354	807	2354	807	ref		1	
4491	32	PC32_13	2002	12-13	12.5	14.2	100 m from Megamat	2354	807	2354	807	ref		1	
4491	32	PC32_15	2002	14-15	14.5	6.6	100 m from Megamat	2354	807	2354	807	ref		1	
4491	32	PC32_17	2002	16-17	16.5	7.6	100 m from Megamat	2354	807	2354	807	ref		1	
4491	29	PC29_1	2002	0-1	0.5	7.5	50 m from Megamat	2403	831	2403	831	no mat		1	
4491	29	PC29_3	2002	2-3	2.5	6.4	50 m from Megamat	2403	831	2403	831	no mat		1	
4491	29	PC29_5	2002	4-5	4.5	9.6	50 m from Megamat	2403	831	2403	831	no mat		1	
4491	29	PC29_7	2002	6-7	6.5	3	50 m from Megamat	2403	831	2403	831	no mat		1	
4491	29	PC29_9	2002	8-9	8.5	7.6	50 m from Megamat	2403	831	2403	831	no mat		1	
4491	29	PC29_12	2002	10-12	11	8.5	50 m from Megamat	2403	831	2403	831	no mat		1	
4491	29	PC29_16	2002	14-16	15	11.2	50 m from Megamat	2403	831	2403	831	no mat		1	
4492	3	PC3_1	2006	0-1	0.5	16.6	Survey site 1	2575	832	2575	832	w	6	3	
4492	3	PC3_3	2006	2-3	2.5	39.6	Survey site 1	2575	832	2575	832	w		3	
4492	3	PC3_5	2006	4-5	4.5	8.9	Survey site 1	2575	832	2575	832	w		3	
4492	3	PC3_7	2006	6-7	6.5	9.5	Survey site 1	2575	832	2575	832	w		3	
4492	3	PC3_9	2006	8-9	8.5	9.6	Survey site 1	2575	832	2575	832	w		3	
4492	3	PC3_11	2006	10-11	10.5	1.4	Survey site 1	2575	832	2575	832	w	50	3	
4492	3	PC3_13	2006	12-13	12.5	1.8	Survey site 1	2575	832	2575	832	w		3	
4492	3	PC3_15	2006	14-15	14.5	5.2	Survey site 1	2575	832	2575	832	w		3	
4492	3	PC3_17	2006	16-17	16.5	1.7	Survey site 1	2575	832	2575	832	w		3	
4492	3	PC3_19	2006	18-19	18.5	5.2	Survey site 1	2575	832	2575	832	w		3	
4492	3	PC3_22	2006	20-22	21.0	16	Survey site 1	2575	832	2575	832	w		3	
4492	4	PC4_1	2006	0-1	0.5	5.4	Survey site 2	2552	738	2552	738	o	7	3	2.4300

4492	4	PC4_3	2006	2-3	2.5	2.7	Survey site 2	2552	738	2552	738	o		3	1.3038
4492	4	PC4_5	2006	4-5	4.5	2.7	Survey site 2	2552	738	2552	738	o		3	0.2113
4492	4	PC4_7	2006	6-7	6.5	1.6	Survey site 2	2552	738	2552	738	o		3	
4492	4	PC4_9	2006	8-9	8.5	6.8	Survey site 2	2552	738	2552	738	o		3	
4492	4	PC4_11	2006	10-11	10.5	2.4	Survey site 2	2552	738	2552	738	o	54	3	
4492	4	PC4_13	2006	12-13	12.5	1.9	Survey site 2	2552	738	2552	738	o		3	
4492	4	PC4_15	2006	14-15	14.5	1.6	Survey site 2	2552	738	2552	738	o		3	
4492	4	PC4_17	2006	16-17	16.5	2.4	Survey site 2	2552	738	2552	738	o		3	
4492	4	PC4_19	2006	18-19	18.5	1.8	Survey site 2	2552	738	2552	738	o		3	
4492	6	bPC6_1	2006	0-1	0.5	6	Survey site 3	2551	897	2551	897	o	4	2	3.6649
4492	6	bPC6_3	2006	2-3	2.5	6.5	Survey site 3	2551	897	2551	897	o		2	2.7035
4492	6	bPC6_5	2006	4-5	4.5	6.4	Survey site 3	2551	897	2551	897	o		2	1.3967
4492	6	bPC6_7	2006	6-7	6.5	3.2	Survey site 3	2551	897	2551	897	o		2	0.5756
4492	6	bPC6_9	2006	8-9	8.5	1.3	Survey site 3	2551	897	2551	897	o		2	0.1848
4492	6	bPC6_11	2006	10-11	10.5	3.8	Survey site 3	2551	897	2551	897	o	27	2	0.2655
4492	6	bPC6_13	2006	12-13	12.5	1.7	Survey site 3	2551	897	2551	897	o		2	0.1332
4492	6	bPC6_15	2006	14-15	14.5	2.9	Survey site 3	2551	897	2551	897	o		2	0.0857
4492	6	bPC6_17	2006	16-17	16.5	5.6	Survey site 3	2551	897	2551	897	o		2	0.1286
4492	6	bPC6_19	2006	18-19	18.5	5.1	Survey site 3	2551	897	2551	897	o	(54)	2	0.0543
4492	6	bPC6_22	2006	21-22	21.5	3.2	Survey site 3	2551	897	2551	897	o		2	0.0429
4492	6	bPC6_24	2006	22-24	23.5	2.4	Survey site 3	2551	897	2551	897	o		2	0.0092
4492	35	bPC35_1	2006	0-1	0.5	27.8	Survey site 1	2575	832	2575	833	w	6	3	
4492	35	bPC35_3	2006	2-3	2.5	9.9	Survey site 1	2575	832	2575	833	w		3	
4492	35	bPC35_5	2006	4-5	4.5	20.5	Survey site 1	2575	832	2575	833	w		3	
4492	35	bPC35_7	2006	6-7	6.5	0.8	Survey site 1	2575	832	2575	833	w		3	
4492	35	bPC35_9	2006	8-9	8.5	2.5	Survey site 1	2575	832	2575	833	w		3	
4492	35	bPC35_11	2006	10-11	10.5	5.9	Survey site 1	2575	832	2575	833	w	50	3	
4492	5	PC5_1	2006	0-1	0.5	43.7	Survey site 2	2552	738	2552	739	w	7	3	
4492	5	PC5_3	2006	2-3	2.5	2.6	Survey site 2	2552	738	2552	739	w		3	
4492	5	PC5_5	2006	4-5	4.5	7.9	Survey site 2	2552	738	2552	739	w		3	
4492	5	PC5_7	2006	6-7	6.5	1.5	Survey site 2	2552	738	2552	739	w		3	
4492	5	PC5_9	2006	8-9	8.5	2.7	Survey site 2	2552	738	2552	739	w		3	
4492	5	PC5_11	2006	10-11	10.5	7.6	Survey site 2	2552	738	2552	739	w	54	3	
4492	7	bPC7_1	2006	0-1	0.5	6	Survey site 3	2551	897	2551	898	y	4	2	
4492	7	bPC7_3	2006	2-3	2.5	7.8	Survey site 3	2551	897	2551	898	y		2	
4492	7	bPC7_5	2006	4-5	4.5	4.1	Survey site 3	2551	897	2551	898	y		2	
4492	7	bPC7_7	2006	6-7	6.5	6.4	Survey site 3	2551	897	2551	898	y		2	
4492	7	bPC7_9	2006	8-9	8.5	4.5	Survey site 3	2551	897	2551	898	y		2	
4492	7	bPC7_11	2006	10-11	10.5	6.9	Survey site 3	2551	897	2551	898	y	27	2	
4492	7	bPC7_13	2006	12-13	12.5	4.5	Survey site 3	2551	897	2551	898	y		2	
4492	7	bPC7_15	2006	14-15	14.5	2.8	Survey site 3	2551	897	2551	898	y		2	
4492	7	bPC7_17	2006	16-17	16.5	0.7	Survey site 3	2551	897	2551	898	y		2	
4493	9	PC9_1	2010	0-1	0.5	11	Survey site 4	2424	683	2424	683	o	4	2	
4493	9	PC9_3	2010	2-3	2.5	2.9	Survey site 4	2424	683	2424	683	o		2	
4493	9	PC9_5	2010	4-5	4.5	2.9	Survey site 4	2424	683	2424	683	o		2	
4493	9	PC9_7	2010	6-7	6.5	1.8	Survey site 4	2424	683	2424	683	o		2	
4493	9	PC9_9	2010	8-9	8.5	2.3	Survey site 4	2424	683	2424	683	o		2	
4493	9	PC9_11	2010	10-11	10.5	3.4	Survey site 4	2424	683	2424	683	o	29	2	
4493	9	PC9_13	2010	12-13	12.5	2.4	Survey site 4	2424	683	2424	683	o		2	
4493	9	PC9_15	2010	14-15	14.5	3.1	Survey site 4	2424	683	2424	683	o		2	
4493	9	PC9_17	2010	16-17	16.5	2	Survey site 4	2424	683	2424	683	o		2	
4493	9	PC9_19	2010	18-19	18.5	4.2	Survey site 4	2424	683	2424	683	o		2	

4493	10	PC10_1	2010	0-1	0.5	25.7	Survey site 4	2424	683	2424	684	o	4	2	
4493	10	PC10_2	2010	1-2	1.5	4.2	Survey site 4	2424	683	2424	684	o		2	
4493	10	PC10_3	2010	2-3	2.5	16.5	Survey site 4	2424	683	2424	684	o		2	
4493	10	PC10_5	2010	4-5	4.5	2.3	Survey site 4	2424	683	2424	684	o		2	
4493	10	PC10_7	2010	6-7	6.5	6.6	Survey site 4	2424	683	2424	684	o		2	
4493	10	PC10_9	2010	8-9	8.5	4.6	Survey site 4	2424	683	2424	684	o		2	
4493	10	PC10_11	2010	10-11	10.5	1.9	Survey site 4	2424	683	2424	684	o	29	2	
4493	10	PC10_13	2010	12-13	12.5	5	Survey site 4	2424	683	2424	684	o		2	
4493	10	PC10_15	2010	14-15	14.5	0.9	Survey site 4	2424	683	2424	684	o		2	
4493	10	PC10_17	2010	16-17	16.5	3.4	Survey site 4	2424	683	2424	684	o		2	
4493	10	PC10_19	2010	18-19	18.5	0.7	Survey site 4	2424	683	2424	684	o		2	
4493	10	PC10_21	2010	20-21	20.5	1.7	Survey site 4	2424	683	2424	684	o	59	2	
4493	14	PC14_1	1995	0-1	0.5	5.8	Survey site 5	2476	768	2476	768	no mat	10	3	1.0435
4493	14	PC14_3	1995	2-3	2.5	1.4	Survey site 5	2476	768	2476	768	no mat		3	0.2330
4493	14	PC14_5	1995	4-5	4.5	1.0	Survey site 5	2476	768	2476	768	no mat		3	0.1420
4493	14	PC14_7	1995	6-7	6.5	0.8	Survey site 5	2476	768	2476	768	no mat		3	0.1200
4493	14	PC14_9	1995	8-9	8.5	1.7	Survey site 5	2476	768	2476	768	no mat		3	0.1165
4493	14	PC14_13	1995	12-13	12.5	4.8	Survey site 5	2476	768	2476	768	no mat		3	
4493	14	PC14_15	1995	14-15	14.5	1.7	Survey site 5	2476	768	2476	768	no mat		3	
4493	14	PC14_17	1995	16-17	16.5	0.2	Survey site 5	2476	768	2476	768	no mat		3	
4493	14	PC14_19	1995	18-19	18.5	0.3	Survey site 5	2476	768	2476	768	no mat		3	
4493	15	PC15_1	2013	0-1	0.5	11.8	Survey site 6	2531	743	2531	743	o	10	2	
4493	15	PC15_3	2013	2-3	2.5	8.7	Survey site 6	2531	743	2531	743	o		2	
4493	15	PC15_5	2013	4-5	4.5	2	Survey site 6	2531	743	2531	743	o		2	
4493	15	PC15_7	2013	6-7	6.5	1.4	Survey site 6	2531	743	2531	743	o		2	
4493	15	PC15_9	2013	8-9	8.5	2.5	Survey site 6	2531	743	2531	743	o		2	
4493	15	PC15_11	2013	10-11	10.5	2.5	Survey site 6	2531	743	2531	743	o	38	2	
4493	15	PC15_13	2013	12-13	12.5	1.6	Survey site 6	2531	743	2531	743	o		2	
4493	15	PC15_15	2013	14-15	14.5	2.4	Survey site 6	2531	743	2531	743	o		2	
4493	15	PC15_17	2013	16-17	16.5	1.2	Survey site 6	2531	743	2531	743	o		2	
4493	15	PC15_19	2013	18-19	18.5	0.9	Survey site 6	2531	743	2531	743	o		2	
4493	16	PC16_1	2013	0-1	0.5	10.2	Survey site 6	2531	743	2531	744	o	10	2	
4493	16	PC16_3	2013	2-3	2.5	15.7	Survey site 6	2531	743	2531	744	o		2	
4493	16	PC16_5	2013	4-5	4.5	16.4	Survey site 6	2531	743	2531	744	o		2	
4493	16	PC16_7	2013	6-7	6.5	3.7	Survey site 6	2531	743	2531	744	o		2	
4493	16	PC16_9	2013	8-9	8.5	15	Survey site 6	2531	743	2531	744	o		2	
4493	16	PC16_11	2013	10-11	10.5	2.7	Survey site 6	2531	743	2531	744	o	38	2	

This table contains the environmental data as it was used in the statistical analyses (grey background). Positional coordinates (on a meter grid scale) are represented by ship- and submersible-fix values and were corrected for some samples to account for the spatial distance between cores taken in the same mat spot. Mat color is coded as follows: w = white mat, o = orange mat, y = yellow mat, ref = reference, no mat = no mat visible. As temperature was not available for all investigated sediment depth layers, 3 categories were defined based on the measured values in the top 10 cm of each core: 1 = cold (<10°C), 2 = medium (10°C≤T<40°C), and 3 = hot (≥40°C). All available temperatures are listed in SI-Table 2. Two cores had no corresponding temperature data, but were defined as belonging to category 1 (cold) based on the fact that they were reference cores (dive 4491, cores 29 and 32). PC14 (dive 4493) was put into T category 3 based on a temperature of 57°C measured at 10 cm depth - the corresponding ARISA sample, though, was removed from all analyses as the concentration of extracted DNA was 0 ng μl^{-1}. Empty cells refer to unavailable data.

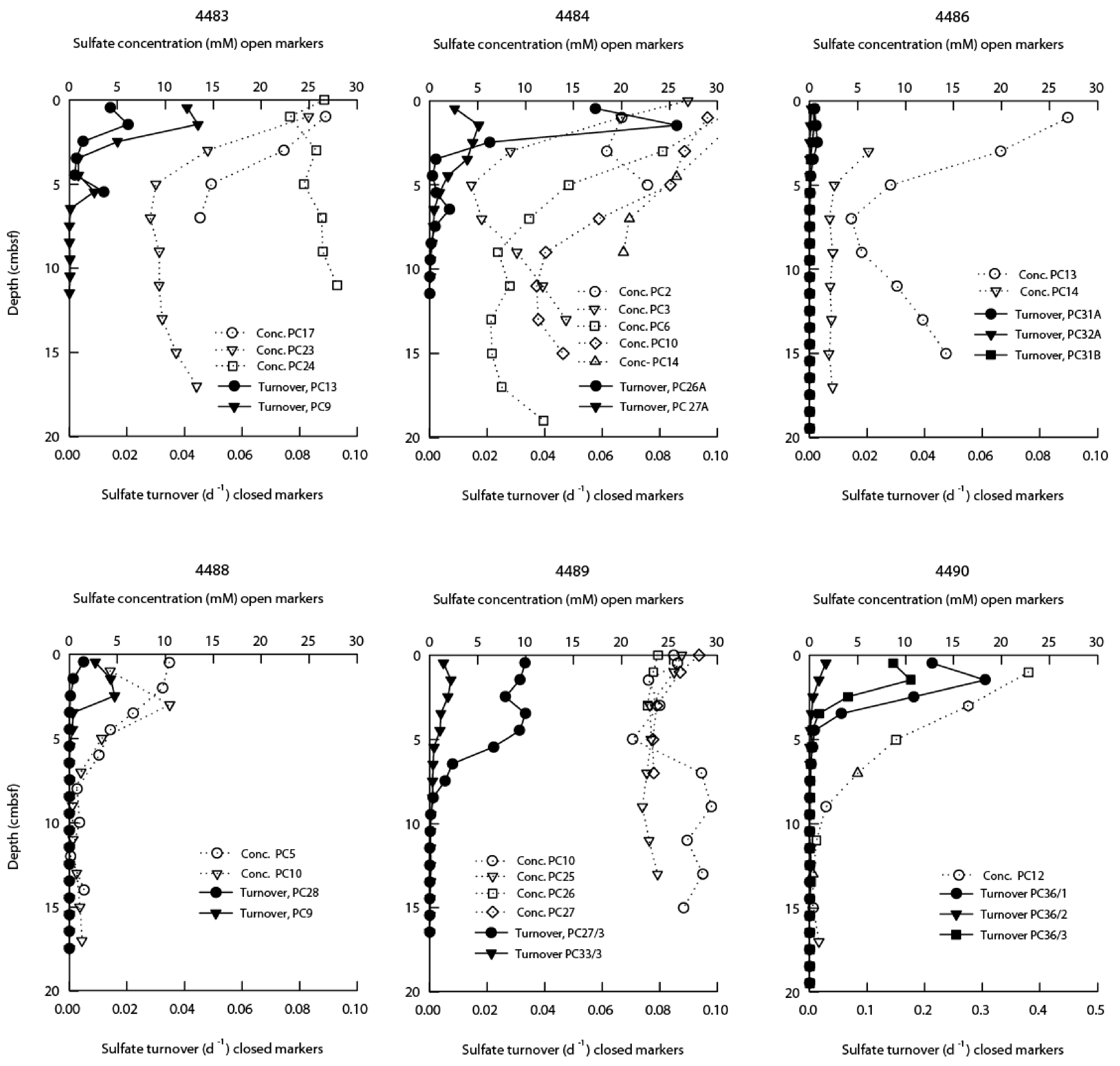

Figure SI-1 – Sulfate concentrations (open markers) and sulfate tracer turnover rate constants (closed markers) at different sampling locations named by Alvin dive. The 4483 and 4484 sediment cores were collected at the base of Mat Mound (Fig. 2A and B). The 4486 and 4488 cores were collected at the edge and in the center of Megamat (Fig. 2C and D). The 4489 cores were collected at UNC Mat, specifically from the orange mat center near the hot AOM core of Biddle et al. 2012 (Fig. 2E and F). The 4490 cores were collected in Megamat, similar to 4488. For each station, cores were collected within a small area. Note the different scale for sulfate turnover in station 4490.

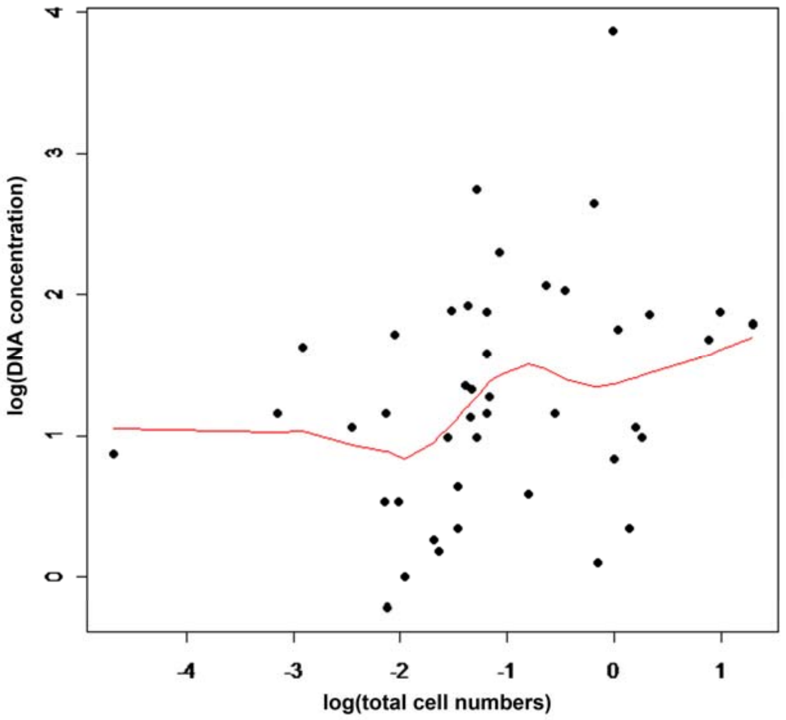

Figure SI-2 – Concentration of extracted DNA as a function of cell number (log-log scale) from Guaymas Basin sediments. The red lines depicts a LOWESS smoother which uses locally-weighted polynomial regression (Cleveland WS 1981, The American Statistician, 35:54).

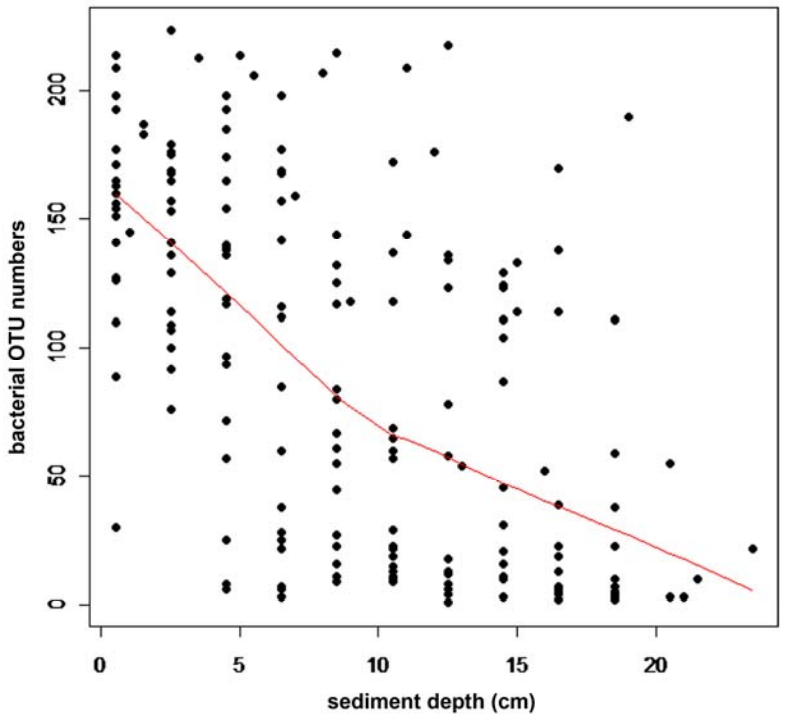

Figure SI-3 – Bacterial OTU numbers determined for Guaymas Basin sediments as a function of sediment depth. The red line represents a LOWESS smoother.

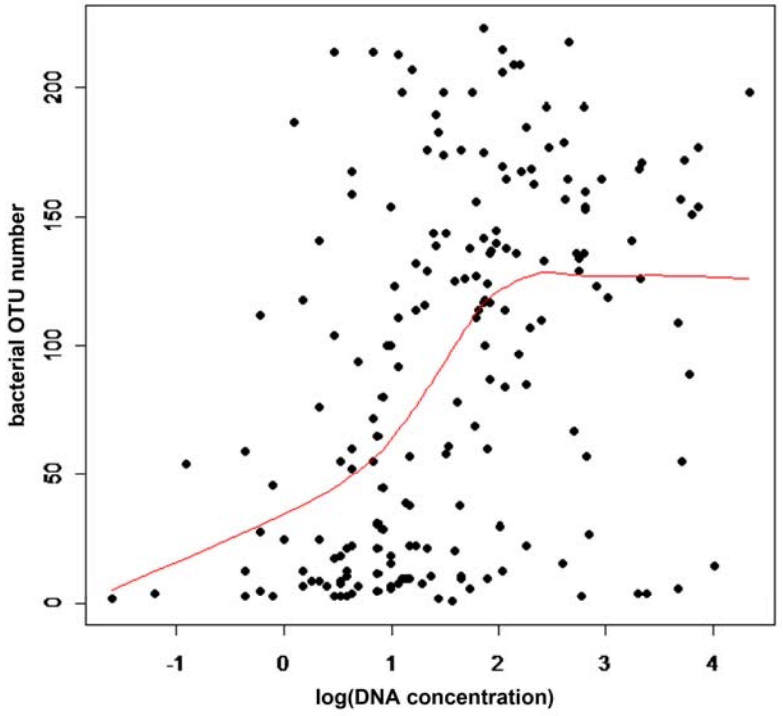

Figure SI-4 – Bacterial OTU numbers determined for Guaymas Basin sediments increased with increasing DNA concentration. The red line represents a LOWESS smoother.

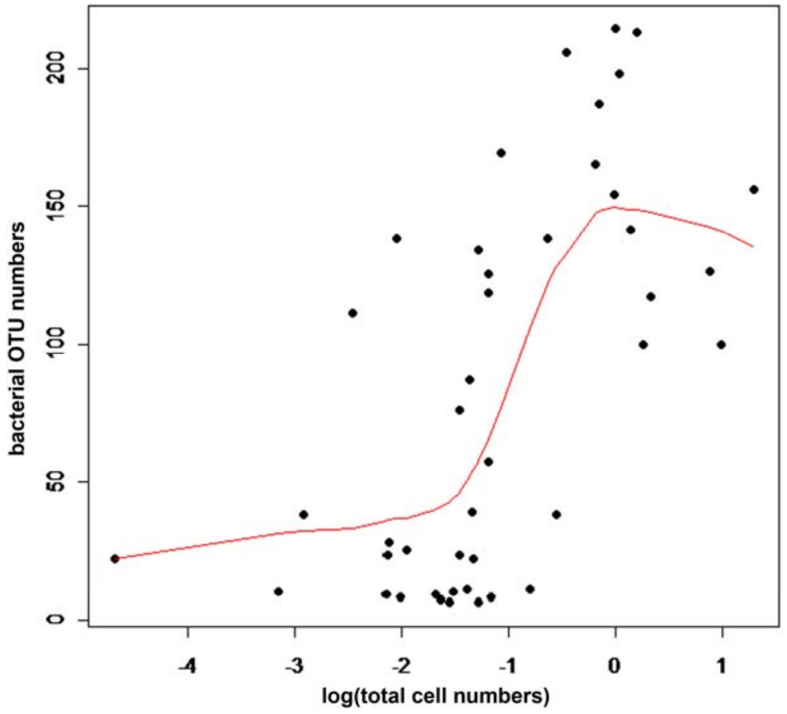

Figure SI-5 – Bacterial OTU numbers in Guaymas Basin sediments generally increased with total cell numbers until a plateau was reached at ~140 OTUs. The red line represents a LOWESS smoother.

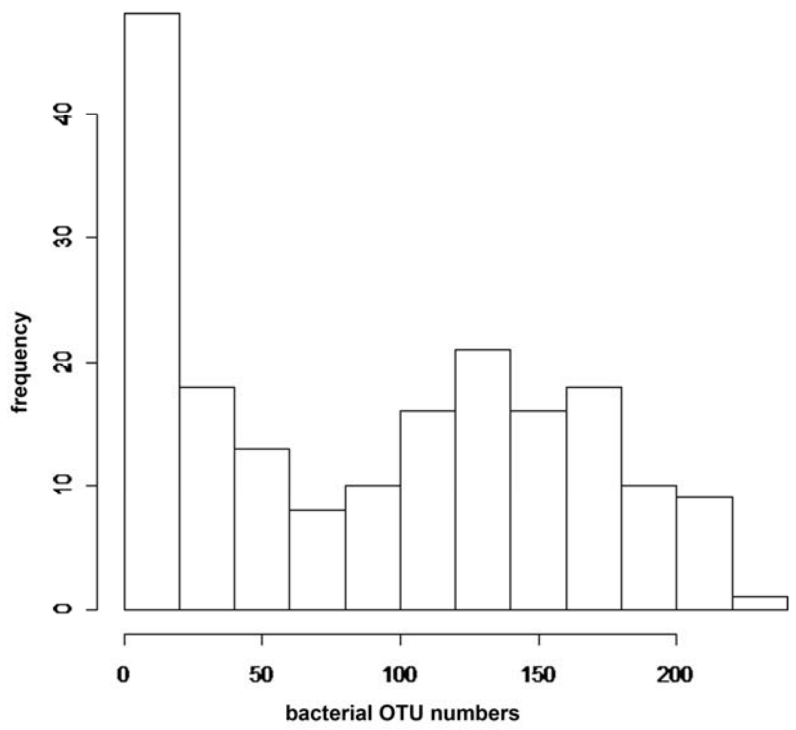

Figure SI-6 – Frequency analysis of Guaymas bacterial OTU numbers.

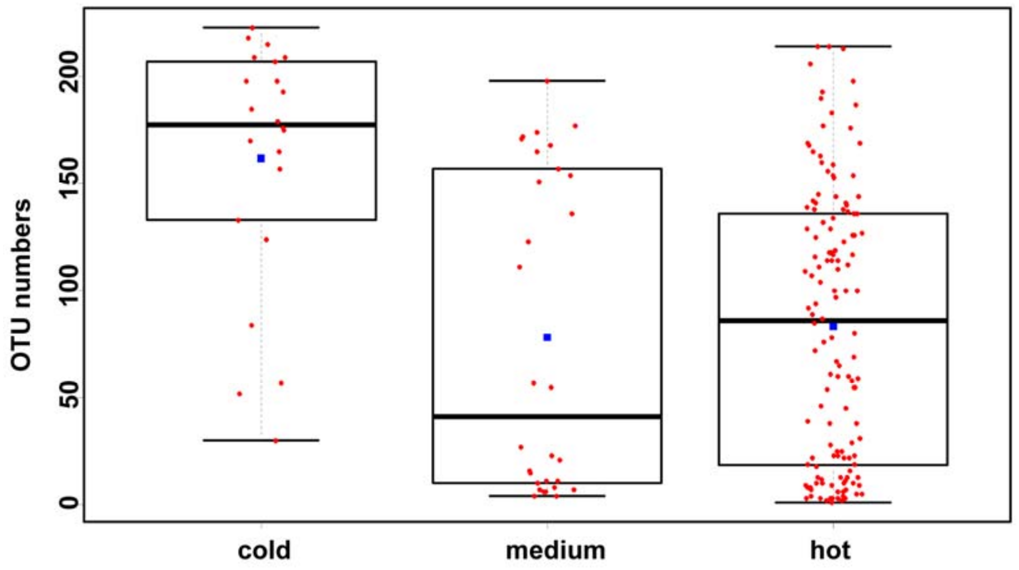

Figure SI-7 – Boxplots representing the variation in OTU numbers as a function of sample temperature classification (cold T<10°C, medium 10°C≤T<40°C, hot T≥40°C, as measured in the upper 10 cm of sediment). Blue dots and thicker lines in the boxes correspond to means and medians, respectively. Red dots correspond to individual measurements with some random noise added to avoid point overlap.
